# Effect of Hydrogen Peroxide on Immersion Challenge of Rainbow Trout Fry with *Flavobacterium psychrophilum*

**DOI:** 10.1371/journal.pone.0062590

**Published:** 2013-04-30

**Authors:** Maya Maria Mihályi Henriksen, Lone Madsen, Inger Dalsgaard

**Affiliations:** Technical University of Denmark, National Veterinary Institute, Bülowsvej 27, Frederiksberg C, Denmark; Beijing Institute of Microbiology and Epidemiology, China

## Abstract

An experimental model for immersion challenge of rainbow trout fry (*Oncorhynchus mykiss*) with *Flavobacterium psychrophilum*, the causative agent of rainbow trout fry syndrome and bacterial cold water disease was established in the present study. Although injection-based infection models are reliable and produce high levels of mortality attempts to establish a reproducible immersion model have been less successful. Various concentrations of hydrogen peroxide (H_2_O_2_) were evaluated before being used as a pre-treatment stressor prior to immersion exposure to *F. psychrophilum*. H_2_O_2_ accelerated the onset of mortality and increased mortality approximately two-fold; from 9.1% to 19.2% and from 14.7% to 30.3% in two separate experiments. Clinical signs observed in the infected fish corresponded to symptoms characteristically seen during natural outbreaks. These findings indicate that pre-treatment with H_2_O_2_ can increase the level of mortality in rainbow trout fry after exposure to *F. psychrophilum*.

## Introduction

*Flavobacterium psychrophilum* is a Gram-negative fish pathogen and the causative agent of bacterial cold water disease (BCWD), also called rainbow trout fry syndrome (RTFS). Since first being discovered in USA in the 1940s, the disease has spread worldwide and was identified in Western Europe in the mid 1980s. Today it has a significant impact on rainbow trout (*Oncorhynchus mykiss*) and other salmonid fish in aquaculture worldwide [1]. Clinical signs of the disease include a dark coloration of the skin, pale gills and organs (kidney, intestine and liver) due to anemia. A loss of appetite and lethargic behavior is also observed.

Injection-based experimental challenges with *F. psychrophilum* have been standardized and the resulting mortality depends on several factors, including the number of colony forming units (CFU) injected, size of the fish, batch differences and the number of fish in each tank [2]. However, infection by injection is not a suitable approach for all investigations since the first line of defense, consisting of non-specific barriers, are bypassed. It has proven difficult to produce high and consistent mortality rates using cohabitation and immersion models unless stress or scarification has been applied [2]–[7], while immersion exposure to *F. psychrophilum* in the logarithmic phase of growth reportedly results in significant mortality rates [8].

Bath-treatment with various non-medical compounds, such as copper sulphate, chloramine-T, sodium carbonates, sodium chloride, formalin and hydrogen peroxide (H_2_O_2_), are routinely used against pathogens in aquaculture [9]. One previous immersion model for *F. psychrophilum* used formalin as stressor, which raised the mortality rate significantly [2]. Since the use of formalin is to be phased out by Danish fish farmers due to human health considerations, another less harmful stressor is needed. H_2_O_2_ is a useful and environmentally friendly alternative to formalin and has been used against skin parasites, bacterial gill infections and mold on eggs in Denmark for over ten years [10], [11]. The use of H_2_O_2_ has been shown to accelerate *Tenacibaculum maritimum* infections in turbot [12] and *Flavobacterium columnaris* infections in channel catfish [13]. Accordingly, H_2_O_2_ was an obvious candidate for a stressor in a *F. psychrophilum* immersion model.

The aim of this study was to establish a reproducible method for increasing mortality of RTFS in immersion challenge of rainbow trout fry. A reliable model is needed for studies on pathogen invasion, host immune response and efficacy of treatments. First, various concentrations of H_2_O_2_ were tested in experiment 1 before being combined with immersion exposure to a virulent *F. psychrophilum* strain at two different phases of bacterial growth in experiment 2. The most favorable H_2_O_2_ concentration and bacterial growth phase were combined in experiment 3, after which the temperature and number of animals were adjusted in experiment 4 to increase the power of the model.

## Materials and Methods

### Fish and Rearing Conditions

Three batches of eggs from the same stock and family of fish were used in all four experiments (see Table 1). The eggs originated from Fousing Trout Farm (Denmark) and disinfection and hatching was carried out at AquaBaltic (Bornholm, Denmark). The fish were acclimatized for at least 3 weeks in 200 L tanks containing 15°C recirculated tap water (non-chlorinated), while nitrate, nitrite and ammonia levels were monitored continuously. The fish were fed dry commercial feed daily (INICIO Plus, BioMar A/S, Denmark). All tanks, except the 1 and 2 L tanks used during challenge and pre-treatment, were opaque in order to minimize external stress due to movement in the experimental facility. Upon arrival, at least 10 fish from each batch where sacrificed by an overdose of MS-222 (Ethyl 3-aminobenzoate methanesulfonate, Sigma), examined for *F. psychrophilum* and proven to be free of the pathogen. Samples from brain and kidney were streaked on tryptone yeast extract salts (TYES) and blood agar plates, while spleen samples were placed in TYES broth (110 rpm). Samples were incubated for 7 d at 15°C for TYES broth/plates and at 20°C for blood plates. Yellow colonies were further examined by species specific PCR. First, a pure culture was obtained by streaking and after 5–7 d of growth, a single colony was suspended in 50 µL distilled water and boiled for 5 min to lyse the bacterial cells and placed on ice. Then, species specific primers, referred to as PSY1 and PSY2, amplifying the 16S rRNA genes of *F. psychrophilum* were used [14] for PCR amplifications, which were carried out according to a previously described method [15]. In brief, Ready to Go PCR beads (cat. no. 27955901, Amersham Pharmacia Biotech, Millwaukee, USA), including all reagents, were used. Ten pmol of each primer and 1 µL of the lysed bacteria were added to each reaction. Distilled water was added to a total volume of 25 µL. Amplifications were carried out using a Biometra T- 3000 thermocycler amplifier (Biometra, Göttingen, Germany): 95°C for 5 min followed by 35 cycles consisting of 30 s at 95°C, 30 s at 57°C and 60 s at 72°C.

**Table 1:** Experimental parameters.

Exp.	Batch	Weight	Length	H_2_O_2_ mg L^−1^	*F.p* CFU mL^−1^	°C	n (pr. replicate)
**1**	1	0.69 g (±0.21)	4.4 cm (±0.3)	50/150/300	–	15.5±1.0	36 (6)
**2**	1	0.77 g (±0.18)	4.4 cm (±0.3)	−/200	10^5^(24 h)/10^7^(48 h)	16.0±0.5	80 (10)
**3**	2	1.2 g (±0.50)	5.0 cm (±0.8)	−/150	−/10^7^	16.8±0.5	400 (50)
**4**	3	1.1 g (±0.34)	4.7 cm (±0.5)	−/200	−/10^7^	14.0±0.5	500 (50)

Fish batches and mean values for weight, length and temperature are stated along with standard deviation (SD). The applied H_2_O_2_ concentrations are given in mg L**^−^** ^1^, while the final bacterial concentrations in broth used for immersion challenge are stated in CFU mL**^−^** ^1^. The symbol ‘−’ designates the absence of *F.p.* and H_2_O_2_.

Before randomly selecting the experimental groups, the largest (approx. >1.5 g) and smallest (approx. <0.6 g) fish were removed in order to minimize size variation. The fish were inspected at least once every 24 h and dead fish were examined for *F. psychrophilum* as previously described. Fish surviving until the completion of each experiment were euthanized using an overdose of MS-222.

The described work was carried out in accordance with the internationally accepted guidelines for care and use of laboratory animals in research and procedures were approved by the Committee for Animal Experimentation, Ministry of Justice, Copenhagen, Denmark (J.nr. 2006/561-1204 and 2011/561-51). During experiments, the fish were inspected several times a day and precautions were taken to minimize stress by keeping the fish in opaque tanks, minimizing handling and monitoring water chemistry.

### Bacterial Strain and Challenge Dose

A Danish *F. psychrophilum* strain, 950106-1/1, was used for all challenges (serotype Fd and elastin degrading). It was isolated from a clinical outbreak of RTFS in a freshwater farm in 1995, and has previously been used for several i.p. and immersion challenges [2]. The bacterial strain was stored at -80°C in TYES broth with 15–20% glycerol and pre-cultivated in 10 mL TYES broth at 15°C for 3 d (110 rpm). Then, 100 mL TYES broth was inoculated using 0.5 mL of the pre-culture and optical density (OD) was measured at 525 nm using a spectrophotometer (Shimadzu UV-1201) at regular intervals to establish a growth curve. Logarithmic and stationary phases were determined after 24 or 48 h of growth, respectively. Before being used for challenge, each culture was examined microscopically to assert purity and CFU counts were carried out by spreading 0.1 mL of 10-fold dilutions (10**^−^** ^4^ to 10**^−^** ^7^) on TYES agar in duplicates.

### Experiment 1: H_2_O_2_ as Pre-treatment Stressor

Three groups of 6 fish in duplicates (n = 36) were immersed for 60 min in aerated 1 L tanks containing final concentrations of 50, 150 or 300 mg H_2_O_2_ L**^−^** ^1^ (prod. no. 1072090250, Merck KGaA, Germany). H_2_O_2_ was added directly into the tanks already containing the fish. Subsequently, the fish were removed and placed in 20 L aquaria and observed for 14 d. The temperature during the experiment was 15±0.5°C, while H_2_O_2_ treatment was carried out at approximately 12°C. Based on the results, a concentration of 200 mg H_2_O_2_ L**^−^** ^1^ was chosen as stressor for experiment 2.

### Experiment 2: H_2_O_2_ Combined with Bacterial Growth Phases (24 and 48 h)

Four groups of 10 fish in duplicates (n = 80) were immersed for 60 min in aerated 1 L tanks containing 200 mg H_2_O_2_ L**^−^** ^1^ or kept under similar conditions in water. H_2_O_2_ was added directly into the aquaria already containing the fish. Afterwards, the fish were removed and placed for 30 min in a tank containing a 1∶9 dilution of cultures grown for either 24 h or 48 h. This resulted in bacterial concentrations of 10^5^ and 10^7^ CFU mL**^−^** ^1^ water, respectively. Finally, the fish were placed in 20 L aquaria containing tap water. The fish were observed for 30 d. The temperature during the experiment was 14.5±0.5°C, while H_2_O_2_ treatment and bacterial challenge were carried out at a temperature below 14°C. The animals dying after exposure to the pathogen were sampled as previously described for the bacteriological examination. Since no significant difference regarding either mortality or reisolation of the pathogen was observed, the 48 h culture was chosen for the following experiments, since it has been used successfully in previous investigations [2].

### Experiment 3∶150 mg L**^−^** ^1^ H_2_O_2_ as Experimental Stressor

Four groups of 50 fish in duplicates (n = 400) were immersed for 60 min in aerated 2 L tanks containing 150 mg H_2_O_2_ L**^−^** ^1^ or kept under similar conditions in tap water. H_2_O_2_ was diluted in 300 mL tap water, before being added to the aquaria already containing the fish. Subsequently, the fish were removed and immersed in a 1∶9 diluted broth culture (48 h) containing 10^7^ CFU mL**^−^** ^1^ for 30 min, while controls were immersed in sterile broth. Finally, the fish were placed in 30 L aquaria and mortality observed for 50 d. The temperature during the experiment was 16.8±0.5°C, while H_2_O_2_ treatment and bacterial challenge were carried out at a temperature below 14°C. To increase mortality in experiment 4, the temperature was lowered and the concentration of H_2_O_2_ increased.

### Experiment 4∶200 mg L**^−^** ^1^ H_2_O_2_ as Experimental Stressor

Four groups of 50 fish in duplicates for uninfected or triplicates for infected (n = 500) were immersed for 60 min in aerated 2 L tanks containing 200 mg H_2_O_2_ L**^−^** ^1^ or kept under similar conditions in tap water. H_2_O_2_ was diluted in 300 mL tap water, before being added to the treatment tanks already containing the fish. Subsequently, the fish were removed and immersed in a 1∶9 diluted broth culture (48 h) containing 10^7^ CFU mL**^−^** ^1^ for 30 min, while controls were immersed in sterile broth. Finally, the fish were placed in 30 L aquaria and observed for 40 d. The temperature during the experiment was 14±0.5°C, while H_2_O_2_ treatment and bacterial challenge were carried out at a temperature below 14°C. Conditions during treatments of the various groups were kept as identical as possible with the same number of nettings and amount of retention time in the 2 L tanks during treatments.

### Statistical Analysis

Mortality data was analyzed using the generalized linear model (GLM) on the R software platform [16] and figures have been produced using GraphPad Prism 5. The relative standard deviation (RSD = (standard deviation/mean)*100) was also calculated for mortality rates of infected groups in experiment 3 and 4. Weight and length of the fish was compared between groups (where a sufficient n was available) by using 1-way ANOVA combined with Tukey’s test.

In tables and figures, significance levels are denoted by * for p = 0.05, ** for p = 0.01 and *** for p = 0.001.

## Results

Hydrogen peroxide pre-treatment was found to elevate the mortality of subsequent immersion exposure to *F. psychrophilum* in the stationary phase of growth.

### Experiment 1: H_2_O_2_ as Pre-treatment Stressor

An apparent escape response was observed in all groups immediately after exposure to H_2_O_2_; the majority of fish clumped together and swam towards the tank wall. This was most prominent in tanks treated with medium and high concentrations, where the escape response was followed by fish laying on bottom of the tank. Furthermore, a few fish from the high dose group showed erratic swimming behavior for shorter periods of time. All visual changes in behavior ceased after a few min at the lowest concentration, while persisting longer in groups treated with higher concentrations of H_2_O_2_. All fish survived the treatment, although one death occurred in the high dosage group within the first 24 h.

### Experiment 2∶200 mg L**^−^** ^1^ H_2_O_2_ Combined with Bacterial Growth Phases (24 and 48 h)

The experiment included four groups in duplicates: (1) 24 h culture, (2) 24 h culture with H_2_O_2_ pre-treatment, (3) 48 h culture and (4) 48 h culture with H_2_O_2_ pre-treatment (Table 2).

**Table 2:** H_2_O_2_ combined with bacterial growth phases.

Group	H_2_O_2_	*F.p*(CFU mL^−1^)	n	Re-isolation/dead fish	% mortality
**1**	–	**24 h**	20	0/3	15%
**2**	+	(10^5^)	12	3/3	25%
**3**	–	**48 h**	20	0/1	5%
**4**	+	(10^7^)	19	3/4	21%

Experiment 2. The symbols ‘+’ and ‘-’ designate addition or absence of the two factors, H_2_O_2_ (200 mg L**^−^** ^1^ for 60 min). and *F.p.* (10^5^/10^7^ CFU mL**^−^** ^1^ *F. psychrophilum* for 30 min). Successful reisolations of *F. psychrophilum* from dead fish are given as a fraction of the total number of dead fish. Finally, cumulative mortality is stated in percent.

Nine of the 40 fish pre-treated with H_2_O_2_ died during treatment and were accounted for as acute mortality. It was only possible to re-isolate *F. psychrophilum* from dead fish belonging to groups pre-treated with H_2_O_2_.

### Experiment 3∶150 mg L**^−^** ^1^ H_2_O_2_ as Experimental Stressor

The experiment consisted of four groups in duplicates: (I) untreated control, (II) H_2_O_2_, (III) *F. psychrophilum* and (IV) H_2_O_2_ and *F. psychrophilum* (Table 3).

**Table 3:** Challenge after 150 mg L**^−^** ^1^ H_2_O_2_.

Group	Replicate	H_2_O_2_	*F.p.*	n	% mortality (n)	Cumulative % mortality	SD
**I**	**A**	–	–	45	2.0% (1)	**2.0%**	
	**B**	–	–	47	2.0% (1)	(a*)	
**II**	**A**	+	–	49	4.1% (2)	**4.0%**	
	**B**	+	–	44	4.4% (2)	(b***)	
**III**	**A**	–	+	49	8.0% (4)	**9.1%**	2.19
	**B**	–	+	27	11.1% (3)	(a*)(c**)	(23%)
**IV**	**A**	+	+	49	14.0% (7)	**19.2%**	7.42
	**B**	+	+	48	24.5% (12)	(b***)(c**)	(39%)

Experiment 3. The fish weighed 1.2 g and the temperature was 16.8±0.5°C. The symbols ‘+’ and ‘-’ designate addition or absence of the two factors, H_2_O_2_ (150 mg L**^−^** ^1^ for 60 min) and *F.p.* (10^7^ CFU mL**^−^** ^1^ *F. psychrophilum* for 30 min). The % cumulative mortality is stated for both replicates and groups along with SD and RSD in brackets. Statistical differences are denoted by a letter and * for p = 0.05, ** for p = 0.01 and *** for p = 0.001.

Seven of the 200 fish pre-treated with 150 mg H_2_O_2_ L**^−^** ^1^ died during the treatment or subsequent transfer to tap water and were accounted for as acute mortality. Seven days after challenge, technical problems lead to an increased temperature in the room and flooding of tank III.B. This led to the loss of 23 fish, which were excluded from the experiment, since the event took place before onset of mortality.

**Figure 1:**
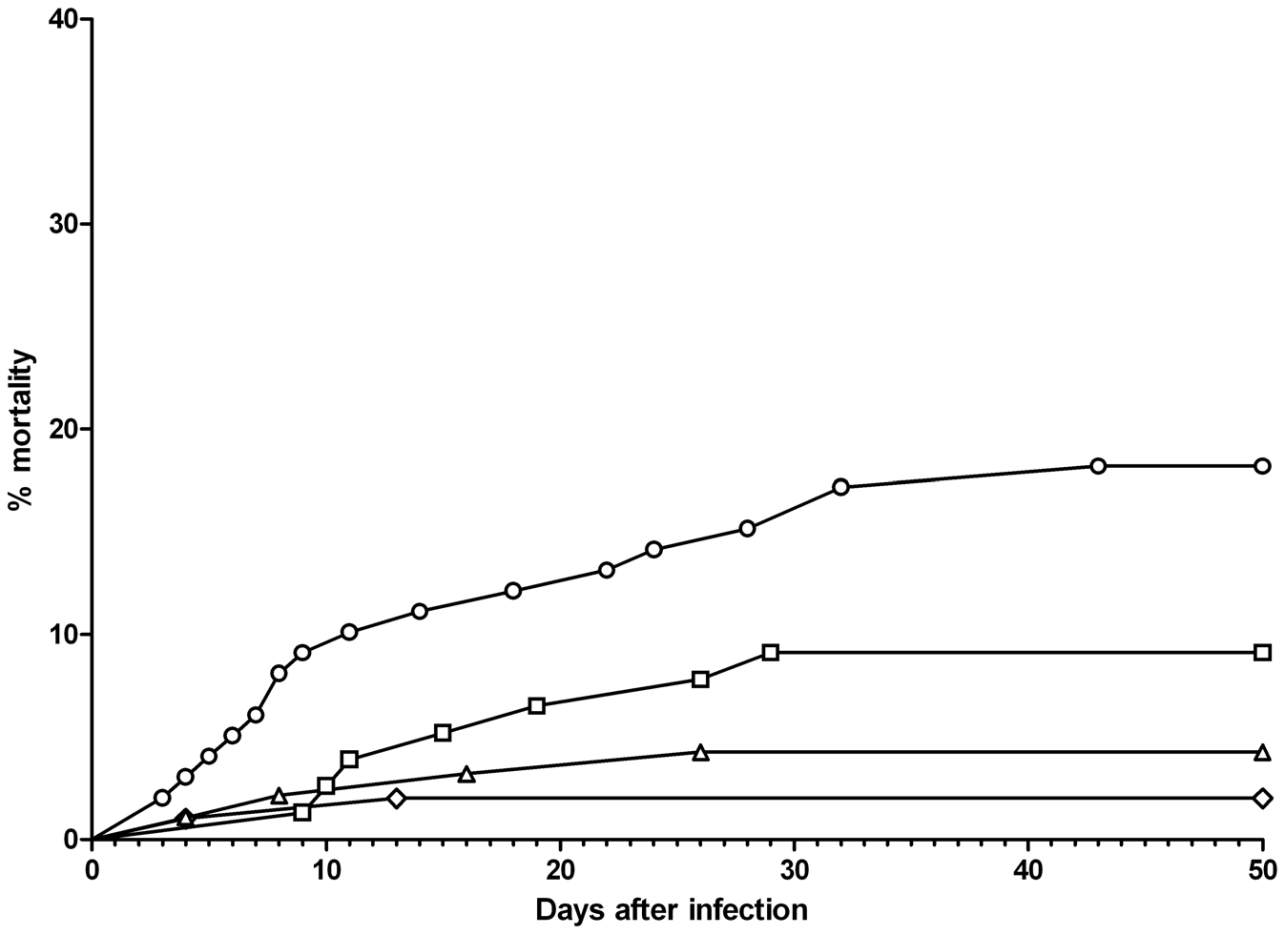
Challenge after 150 mg L^−1^ H_2_O_2_. Cumulative mortality of the merged replicates is shown in the figure. The fish weighed 1.2 g and the temperature was 16.8±0.5°C. The experiment ran for 50 days. **⋄:** control, **▵:** H_2_O_2_, **□:** *F. psychrophilum*, **○:** H_2_O_2_+ *F. psychrophilum*.

The cumulative mortality (Table 3) was higher in both infected groups compared to their respective controls (p = 0.05 for III and p = 0.001 for IV). The cumulative mortality for infected groups was 9.1% for *F. psychrophilum* challenge alone and 19.2% in combination with H_2_O_2_ (p = 0.001). Relative standard deviation for the two groups was 23% and 39%, respectively. Furthermore, H_2_O_2_ accelerated the onset of mortality post challenge from 9 to 3 d (Fig. 1).

### Experiment 4∶200 mg L**^−^** ^1^ H_2_O_2_ as Experimental Stressor

The experiment consisted of four groups, which were set up in either duplicates for uninfected or triplicates for infected groups: (A) untreated control and (B) H_2_O_2_, (C) *F. psychrophilum* and (D) H_2_O_2_ and *F. psychrophilum* (Table 4).

**Table 4:** Challenge after 200 mg L**^−^** ^1^ H_2_O_2_.

Group	Replicate	H_2_O_2_	*F.p.*	n	% mortality (n)	Cumulative % mortality	SD
**A**	**1**	–	–	50	0.0% (0)	**3%**	
	**2**	–	–	50	6.0% (3)	(a***)	
**B**	**1**	+	–	50	8.0% (4)	**5%**	
	**2**	+	–	50	2.0% (1)	(b***)	
**C**	**1**	–	+	50	12.0% (5)	**14.7%**	4.19
	**2**	–	+	50	14.3% (7)	(a***)(c***)	(27%)
	**3**	–	+	50	20.0% (10)		
**D**	**1**	+	+	50	20.0% (10)	**30.3%**	11.57
	**2**	+	+	49	42.9% (21)	(b***)(c***)	(39%)
	**3**	+	+	46	28.3% (13)		

Experiment 4. The fish weighed 1.1 g and the temperature was 14±0.5°C. The symbols ‘+’ and ‘–’ designate addition or absence of the two factors, *F.p.* (10^7^ CFU mL**^−^** ^1^ *F. psychrophilum* for 30 min) and H_2_O_2_ (200 mg L**^−^** ^1^ for 60 min). The % cumulative mortality is stated for both replicates and groups along with SD and RSD. Statistical differences are denoted by a letter and * for p = 0.05, ** for p = 0.01 and *** for p = 0.001.

Five of the 250 fish pre-treated with 200 mg H_2_O_2_ L**^−^** ^1^ died during the treatment and subsequent transfer to tap water and were accounted for as acute mortality.

**Figure 2:**
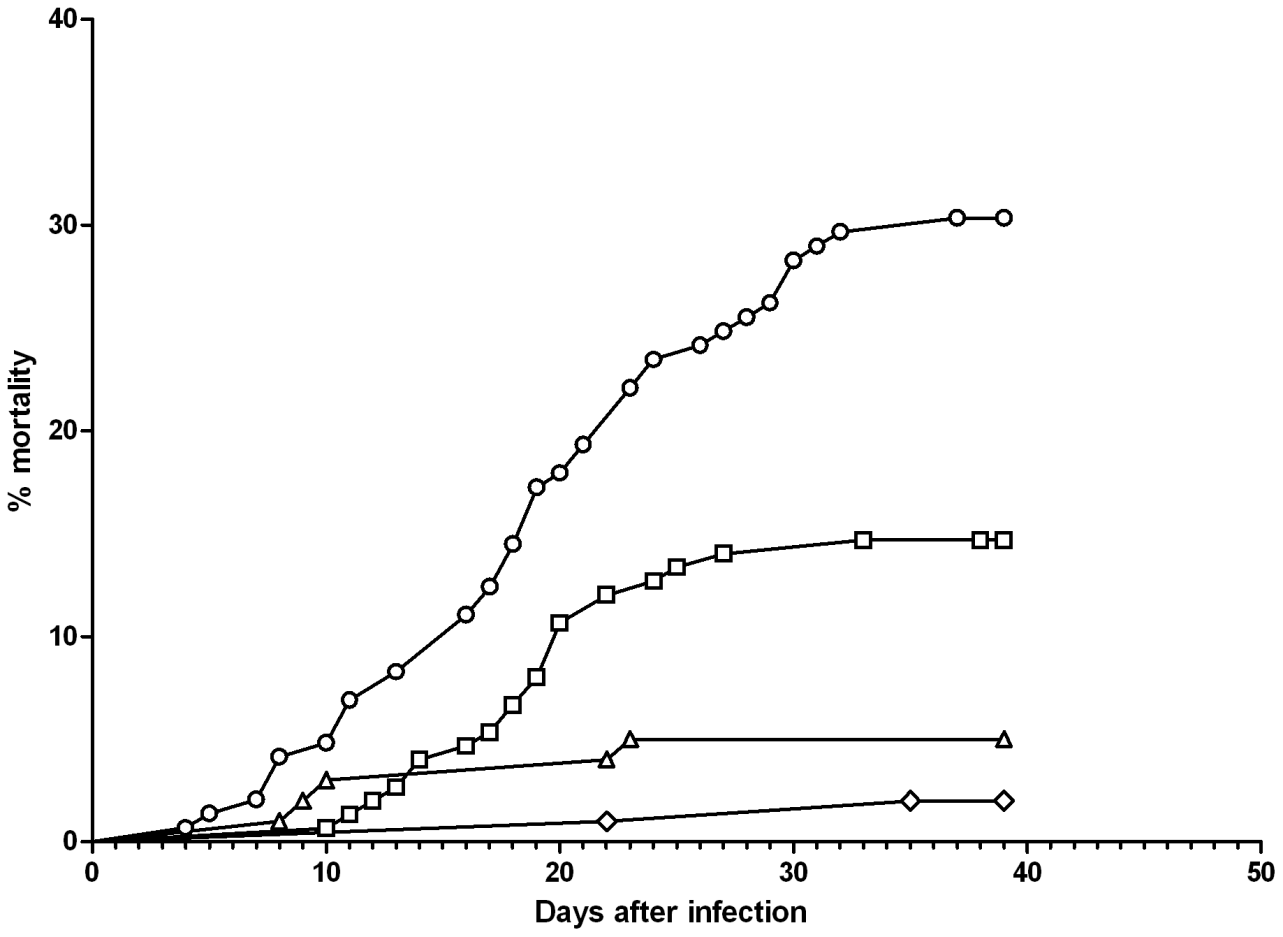
Challenge after 200 mg L^−1^ H_2_O_2_. Cumulative mortality of the merged replicates is shown in the figure. The fish weighed 1.1 g and the temperature was 14±0.5°C. The experiment ran for 40 days. **⋄:** control, **▵:** H_2_O_2_, **□:** *F. psychrophilum*, **○:** H_2_O_2_+ *F. psychrophilum*.

The cumulative mortality was higher in both infected groups compared to their respective controls (p = 0.001). The cumulative mortality (Table 4) of infected groups was 14.7% (n = 22) for *F. psychrophilum* challenge alone and rose to 30.3% (n = 54) in combination with H_2_O_2_ (p = 0.001). RSD for the two groups was 27% and 38%, respectively. Furthermore, H_2_O_2_ accelerated the onset of mortality post challenge from 10 to 4 d (Fig. 2). A statistically significant difference in mortality was found between replicate tanks D1 (20.0%) and D2 (42.9%) from group D (p = 0.05), which was taken into consideration in data processing.

## Discussion

Several studies have investigated the potential adverse effects of H_2_O_2_ on salmonids to establish recommendations for safe treatment dosages [10], [11], [17]–[20]. Possible damage caused by H_2_O_2_ depends on several factors, including the applied concentration, exposure time, frequency of treatment, life stage of the fish and temperature during treatment. Damage generally consists of injury to the gills and larger fish are more susceptible [19]. Pathological changes may include an increase in epithelial cell granularity, edemas, lamellar fusion, epithelial hyperplasia as well as swelling and lifting of the gill epithelium. The gills are a frequent target in stress responses and of the mentioned lesions can, with the existing knowledge, be induced by many types of environmental stressor; not just H_2_O_2_ [23]. Treatment is generally discouraged at temperatures above 14–15°C [11], [21], [22], but temperatures approaching 14°C during H_2_O_2_ treatment did not result in increased mortality under the conditions applied in the present experiments.

Mortality due to H_2_O_2_ has been shown to occur within the first days after treatment; predominantly during or within hours of exposure [17], [18]. It is difficult to directly compare the published studies regarding H_2_O_2_, and the performance of pilot studies on small groups of fish are recommended before treating an entire population [18], [19].

### H_2_O_2_ as Pre-treatment Stressor

Previous studies have demonstrated that H_2_O_2_ has a fast short-term stress effect in both Atlantic salmon and sea bass [24], [25]. In the present study, an escape response was observed in all fish treated with H_2_O_2_, although most prominent in the high dose group, in which the only mortality occurred within 24 h after exposure. Based on these results, a concentration of 200 mg H_2_O_2_ L**^−^** ^1^ was chosen as stressor for experiment 2, where 9 of the 40 treated fish died from the pre-treatment alone. The increased mortality is likely due to the direct administration of undiluted 35% H_2_O_2_ into the water. Since the density of fish was higher in experiment 2, the probability of contact with high local concentrations of H_2_O_2_ before dispersal in the water was increased. In experiment 3 and 4, H_2_O_2_ was diluted at in least 300 mL water before being added to the treatment tanks. This approach resulted in 2–3.5% acute mortality in addition to 4–5% mortality during the experiments, compared to a total of 2–3% in the untreated control groups. In a previous test, healthy juvenile rainbow trout were treated twice a week for seven weeks (200 mg L**^−^** ^1^ for 60 min) reported lower feed conversion ratios in the first weeks of treatment but no noteworthy mortality [26], while the same dosage given only once resulted in high mortalities within a few hours under slightly different conditions [17] . The experimental design was not intended to investigate the potential long-term consequences of H_2_O_2_ treatment, but the results indicate the possibility of excess mortality due to as little as a single treatment but the subject should be investigated further.

### H_2_O_2_ Combined with Bacterial Growth Phases (24 and 48 h)

Experiment 2 was a pilot test used to indicate, whether there was a difference in mortality for fish infected with bacteria in either the logarithmic or stationary phase of growth. Furthermore, it was relevant to conduct a small scale test of H_2_O_2_ in combination with *F. psychrophilum*. Based on previous results, an increased mortality was expected for the 24 h culture [8], but no differences were seen. Reisolation of *F. psychrophilum* from spleen, kidney and brain of dead fish was only successful in H_2_O_2_ treated groups. Although the portals of entry have not been determined, *F. psychrophilum* has been found in mucus, fins, gills and stomach of infected fish and it has been speculated, that sub-optimal environmental conditions may allow the bacterium to get across skin and gills [27]. Besides stressing the host, treatment with H_2_O_2_ may have resulted in better access to the blood stream, possibly via the gills or by damaging mucus on either skin or gastrointestinal tract, hence allowing for a rapid spread inside the host.

Based on these findings, it was decided to proceed using H_2_O_2_ as a stressor and the 48 h culture. The 48 h culture has been used successfully in previous investigations [2] and resulted in a higher number of CFU. Cumulative mortality was relatively low for all treatments in experiment 2. The small size of the experimental groups could have affected the outcome in several ways and the results of this experiment cannot be considered to be conclusive. Firstly, the dynamics between healthy, infected and dead fish were influenced by the low density. Secondly, the low number of fish also decreases the statistical robustness, especially since reproducibility is a known problem regarding immersion challenge with *F. psychrophilum*. Choice of strain might have played a role, although 950106-1/1, which was used in the present study, is known to cause mortality in rainbow trout fry [2].

### H_2_O_2_ as Experimental Stressor in Immersion Challenge

The cumulative mortality was significantly increased by pre-treatment with H_2_O_2_ in both experiment 3 and 4. In experiment 3, pre-treatment with H_2_O_2_ increased mortality due to *F. psychrophilum* from 9.1% to 19.2%. In experiment 4, the temperature was lowered and the pre-treatment dosage increased slightly, resulting in mortality rates of 14.7% for *F. psychrophilum* alone and 30.3% in combination with H_2_O_2_. The increased dosage of H_2_O_2_ may have stressed the fish more or resulted in more damage to gills or other tissues in contact with the surrounding water, such as gastrointestinal tract or skin. The lowered temperature is also likely to have played a role, since even smaller changes have been shown to have significant consequences on survival of infections [27]. At lower temperatures, the immune response of the fish is delayed [28], [29], while the psychrophilic bacteria’s physiological functions are impaired to a lesser degree [1]. Another explanation is a suppression of the immune system due to cortisol, which is a corticosteroid associated with stress [23]. Finally, batch differences can also have contributed to the difference in mortality seen between the two experiments.

Variation was consistent around 40% for the pre-treated and infected group, and 25% for the infected group for both experiment 3 and 4. Thus, variation did not seem to be influenced by smaller differences in handling through the experiments, since strictly uniformed management was applied in experiment 4. Both variation and proportional change in mortality induced by H_2_O_2_ was comparable between the two experiments and to the results obtained in a similar model using formalin [2]. Despite the variation, H_2_O_2_ pre-treatment consistently elevated the mortality after subsequent immersion exposure to *F. psychrophilum*. Conversely, the results underline the importance of maximizing the statistical power of the experimental model by using larger experimental groups or more replicates.

Onset of mortality was accelerated, a pattern which was also observed for H_2_O_2_ treatment in combination with *T. maritimum* and *F. columnaris* [12], [13]. The clinical signs in both experiment 3 and 4 corresponded to the symptoms seen in natural outbreaks and included anemia (resulting in pale gills and organs), splenomegaly (which could in some cases be seen through the skin as a red coloration of the abdomen) and dark pigmentation of the skin [1], [30], [31].

A number of previous studies have also focused on developing a reliable model for infection, which mimics natural transmission, since it is essential to gain more knowledge regarding transmission and host-pathogen interactions. Previous studies have highlighted the limited pathogenicity of *F. psychrophilum* without using various forms of stress or scarification. A number of studies have not resulted in mortality [4], [5], [32], [33], while infections have been successfully established in other studies [2], [7], [8], [34]. Even when mortality occurred, reproducibility has been a problem [35].

It is hard to directly compare the various studies due to differences in setup; including origin, size and health status of the fish, differences in bacterial strains and growth conditions, method of exposure and diversity in the experimental setup, including the number replicates and animals in each group. Since it is difficult to induce *F. psychrophilum* mortality without using stressors or compromising the outer barriers, it seems unlikely that immersion challenge will be standardized on the same level as seen with injections. Although mortality did not reach 60%, which is the desired goal for testing the potency of fish vaccines [36], the current model seems reproducible, since mortality was elevated in both full scale experiments.

In conclusion, the investigated model seems to be a good alternative to injections for studies requiring natural transmission of the pathogen without bypassing the outer barriers. Although the present study does not deal with the cause for the increased mortality induced by H_2_O_2_, it emphasizes and allows further investigation of the potential connection between routine non-medical treatments and pathogen outbreaks in aquaculture.
